# The periosteal requirement and temporal dynamics of BMP2‐induced middle phalanx regeneration in the adult mouse

**DOI:** 10.1002/reg2.81

**Published:** 2017-08-20

**Authors:** Lindsay A. Dawson, Ling Yu, Mingquan Yan, Luis Marrero, Paula P. Schanes, Connor Dolan, Maegan Pela, Britta Petersen, Manjong Han, Ken Muneoka

**Affiliations:** ^1^ Veterinary Physiology and Pharmacology Texas A&M University 4466 TAMU College Station TX USA; ^2^ Cell and Molecular Biology Tulane University 2000 Percival Stern Hall, 6400 Freret St New Orleans LA USA; ^3^ Department of Medicine Louisiana State University Health Sciences Center New Orleans LA USA

**Keywords:** BMP2, digit, endochondral ossification, periosteum, regeneration

## Abstract

Regeneration of mammalian limbs is restricted to amputation of the distal digit tip, the terminal phalanx (P3). The adjacent skeletal element, the middle phalanx (P2), has emerged as a model system to investigate regenerative failure and as a site to test approaches aimed at enhancing regeneration. We report that exogenous application of bone morphogenetic protein 2 (BMP2) stimulates the formation of a transient cartilaginous callus distal to the amputation plane that mediates the regeneration of the amputated P2 bone. BMP2 initiates a significant regeneration response during the periosteal‐derived cartilaginous healing phase of P2 bone repair, yet fails to induce regeneration in the absence of periosteal tissue, or after boney callus formation. We provide evidence that a temporal component exists in the induced regeneration of P2 that we define as the “regeneration window.” In this window, cells are transiently responsive to BMP2 after the amputation injury. Simple re‐injury of the healed P2 stump acts to reinitiate endogenous bone repair, complete with periosteal chondrogenesis, thus reopening the “regeneration window” and thereby recreating a regeneration‐permissive environment that is responsive to exogenous BMP2 treatment.

## 1 INTRODUCTION

Salamanders display astonishing regenerative ability whereas mammals, such as mice and humans, appear resistant to regeneration, responding to amputation injury only by wound repair and scar formation. Closer analysis reveals that mice and humans do indeed have regenerative capabilities, but these are restricted to portions of the distal digit tip, the terminal phalanx (P3) (Borgens, 1982; Douglas, 1972; Illingworth, 1974). The regeneration response is amputation‐level‐specific. Amputation levels proximal to the P3 nail matrix fail to elicit a significant regeneration response, resulting in bone truncation and soft tissue scar formation. In contrast, distal level amputations result in the restoration of lost structures (Agrawal, Kelly et al., 2011; Agrawal, Tottey et al., 2011; Agrawal et al., 2010; Dawson et al., 2016; Fernando et al., 2011; Han, Yang, Lee, Allan, & Muneoka, 2008; Masaki & Ide, 2007; Miura, Takahashi, Satoh, & Endo, 2015; Mu, Bellayr, Pan, Choi, & Li, 2013; Neufeld, 1985, 1989; Takeo et al., 2013; Yu, Han, Yan, Lee, & Muneoka, 2012; Yu, Han et al., 2010). At the core of the mammalian regeneration response is the formation of the blastema, the fundamental structure that distinguishes epimorphic regeneration from tissue repair and subsequent scarring (Simkin, Sammarco, Dawson, Schanes et al., 2015). The mammalian P3 blastema is defined as an avascular heterogeneous population of stem and lineage‐restricted proliferating cells formed distal to the amputation stump, that functions to regenerate the missing bone as well as the associated soft connective tissues (Fernando et al., 2011; Han et al., 2008; Lehoczky, Robert, & Tabin, 2011; Rinkevich, Lindau, Ueno, Longaker, & Weissman, 2011; Takeo et al., 2013). This regeneration response involves a sequence of events including inflammation and tissue histolysis, wound closure and blastema formation, and redifferentiation of bone via intramembranous ossification, and serves as a mammalian model for successful epimorphic regeneration (Fernando et al., 2011; Simkin, Sammarco, Dawson, Schanes et al., 2015; Simkin, Sammarco, Dawson, Tucker et al., 2015).

Amputation of the middle phalanx (P2) is regeneration‐incompetent and has emerged as a system to investigate regenerative failure and as a site to test approaches aimed at enhancing regeneration (Agrawal, Kelly et al., 2011; Agrawal, Tottey et al., 2011; Agrawal et al., 2010; Dawson et al., 2016; Miura et al., 2015; Mu et al., 2013; Yu et al., 2012). Comparable to other long bones, P2 amputation results in bone truncation and skin healing with fibrotic scar formation. Recent studies have shown that, despite the absence of a regenerative response, P2 amputation initiates a dynamic repair response similar to fracture healing and displays signs of soft tissue regeneration (Dawson et al., 2016). This finding is important since mammalian bones typically respond to fracture injury by successfully regenerating new bone to bridge the fracture (Einhorn, 2005; Shapiro, 2008). The P2 bone responds to amputation by forming a peripheral cartilaginous callus that is derived from the periosteum, osteoblast recruitment to form woven bone, and remodeling of the woven bone into a lamellar structure that resembles the original bone tissue (Dawson et al., 2016). This is a highly dynamic response that increases local stump bone volume only to remodel it back to the original stump morphology. We hypothesize that this repair response is analogous to a failed attempt at bone regeneration.

It is well documented that bone morphogenetic protein (BMP) signaling is required for endogenous digit tip regeneration (Han, Yang, Farrington, & Muneoka, 2003; Yu, Han et al., 2010). Indeed, the local application of BMP2 induces level‐specific regeneration of neonatal digit amputations (Lee et al., 2013; Yu et al., 2012) as well as regeneration of neonatal and adult limb amputations (Ide, 2012; Masaki & Ide, 2007; Yu et al., 2012). For induced neonatal digit regeneration, a microcarrier bead is used for targeted application of BMP2 that stimulates the formation of an endochondral ossification center that organizes the patterned ossification response (Yu et al., 2012). The BMP2 response in limb amputations is less well characterized, in part because the amputated limb stump is large and an adequate BMP2 vehicle has not been identified. Urist originally identified BMP2 as an agent that induced ectopic bone formation when implanted in vivo (Reddi & Huggins, 1972; Urist, 1965; Wozney et al., 1988). More recently, BMPs have been shown to act redundantly during skeletal development, functioning early in mesenchymal cell condensation and later as a requirement for endochondral ossification (Bandyopadhyay et al., 2006; Barna & Niswander, 2007). In adults, BMP2 is important in the early periosteal response to fracture (Tsuji et al., 2006; Wang, Huang, Xue, & Zhang, 2011), and is required for cell differentiation during the many stages of fracture healing (Minear, Leucht, Miller, & Helms, 2010; Wang et al., 2011; Yu, Lieu et al., 2010). Together, these studies demonstrate that BMP signaling plays a critical role in both skeletal development and endogenous skeletal repair and regeneration, and can be used as a treatment to enhance skeletal regeneration.

In this study, P2 level amputation of the adult mouse digit is induced to regenerate by treatment with a BMP2 slow release vehicle. The induced regeneration response was characterized in detail using quantitative micro‐computed tomography (μCT), histology, and immunohistochemistry. BMP2 treatment induced endochondral ossification at the amputation site that completely restored the amputated P2 bone length, but not the joint or the distal P3 skeletal element. The regenerated bone integrates with the P2 stump and tendon repair was observed. Histologically, BMP2‐induced regeneration is mediated by the formation of a distal chondrogenic callus that undergoes subsequent ossification. Distal callus formation requires the stump periosteum and the formation of the peripheral chondrogenic callus that forms during the amputation response (Dawson et al., 2016). The responsiveness to BMP2 is transient suggesting a dynamic wound environment that we have defined as the “regeneration window.” The healing of long‐term amputations results in a stump that is refractory to BMP2 treatment; however, re‐injury of the P2 stump reinitiates the formation of the peripheral callus as well as the responsiveness to BMP2 treatment. These studies identify and characterize a mechanism of BMP2‐induced regeneration following digit amputation in adult mice.

## 2 RESULTS

### 2.1 BMP2‐induced P2 regeneration

**Figure 1:**
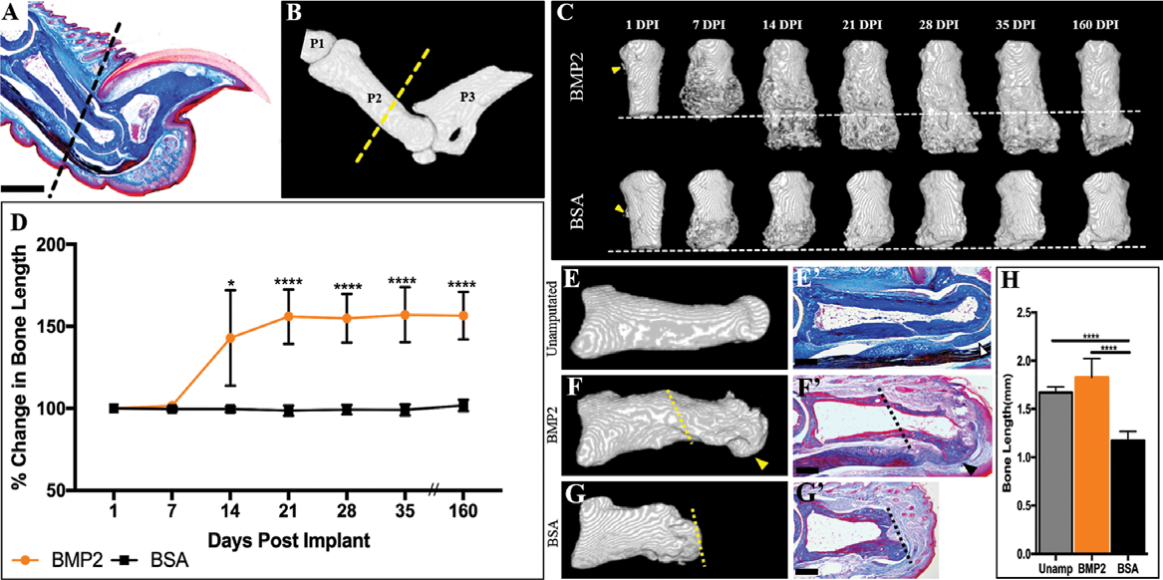
Exogenous BMP2 induces P2 regeneration. (A) Mallory trichrome stained section illustrating the anatomy of the adult mouse hindlimb digit. (B) μCT 3D rendering of the adult mouse hindlimb digit, showing the first (P1), middle (P2), and terminal phalanx (P3). (A), (B) The dashed line indicates the amputation plane. (C) Representative μCT renderings show that BMP2 induces segment‐specific regeneration of P2 via formation of the distal callus, evident by 14 DPI, while BSA‐treated digits do not show distal bone elongation. The amputation plane is shown as the dashed line. (D) Bone length measurements over 160 days, normalized to 1 DPI, illustrate a significant increase in bone length after BMP2 treatment, resulting in an average 56% enhancement of length by 160 DPI, while BSA treatment does not induce distal bone regeneration (*t* test, ±SEM, *P ≤ 0.05, ****P ≤ 0.0001). (E), (E′) Representative μCT 3D rendering and Mallory trichrome stained histological section of the unamputated P2 digit. Open arrowhead denotes ventral tendon and associated fibrocartilage. (F), (F′) Representative μCT 3D rendering and corresponding Mallory trichrome stained histological section of a BMP2‐treated regenerated P2 bone at 160 DPI. Arrowheads indicate the region of distal bone end curvature and associated ventral tendon attachment sites. The amputation plane is shown as the dashed line. (G), (G′) Representative μCT 3D rendering and corresponding Mallory trichrome stained histological section of the BSA‐treated digit at 160 DPI. The amputation plane is shown as the dashed line. (H) BMP2 treatment restores the bone length of amputated P2 digits (ANOVA, ±SEM, *P* ≤ 0.0001). (A), (B), (E)–(G′) Distal is to the right, dorsal is to the top. (C) Distal is to the bottom. Scale bars: (A), (E), (F′) 500 μm; (G′) 200 μm

Amputation of the middle phalanx (P2) is a model system to investigate regenerative failure as well as induced regeneration of the skeletal element and associated soft tissues (Agrawal et al., 2011; Agrawal et al., 2011; Agrawal et al., 2010; Dawson et al., 2016; Miura et al., 2015; Mu et al., 2013; Yu et al., 2012). Digit amputation transects the P2 diaphysis, the dorsal ligament, the skin and associated structures including the dermis and hair follicles, and the ventral fibrocartilage and associated tendon (Fig. 1A, B). In neonatal mice, targeted BMP2 treatment using a microcarrier bead vehicle after wound closure induced a P2 segment‐specific regenerative response (Yu et al., 2012). Using a similar approach but with a slow release vehicle, we implanted BMP2 (*n* = 5) or bovine serum albumin (BSA) (*n* = 5) containing XeroGel at the time of wound closure (9 days post amputation [DPA]) between the wound epidermis and stump bone. At the time of treatment, the amputated P2 stump bone displayed a lateral periosteal chondrogenic callus and the digit stump was capped distally by a wound epidermis (Dawson et al., 2016). Experimental (BMP2 XeroGel) and control (BSA XeroGel) treated digits were followed using μCT imaging for 160 days and changes in phalangeal length and anatomy were quantified (Fig. 1C). μCT 3D rendered images at 1 day post implantation (DPI) show no gross anatomical differences between BMP2 and BSA treatment, with both displaying periosteal remodeling (Fig. 1C, arrowheads). By 7 DPI, the stump bones from both treatment groups show evidence of peripheral callus formation associated with the periosteum (Fig. 1C). The peripheral callus typically forms following simple P2 amputation (Dawson et al., 2016). By 14 DPI, four of five BMP2‐treated digits show the formation of a boney distal callus that is contiguous with the stump bone and extends the length of the P2 element (Fig. 1C). The distal regeneration of bone results in a significant increase in bone length compared to BSA control digits (Fig. 1D, *P* ≤ 0.05). The lengthening of the bone stump is completed by 21 DPI (Fig. 1D, *P* ≤ 0.0001), with bone length maintained over the course of the study.

We performed end‐point comparative analysis of the unamputated P2 bone, BMP2‐treated digits, and BSA‐treated digits at 160 DPI using μCT and histology. The unamputated P2 bone exhibits a defined bulbous distal joint region of articular cartilage that is enclosed by a ventral tendon that inserts to the P3 bone, and ligament positioned dorsally (Fig. 1E, E′, ventral tendon shown as open arrowhead). In BMP2‐treated digits, the regenerated distal bone exhibits bulbous curvature, yet there is no histological evidence of articular cartilage regeneration (Fig. 1F, arrowhead). μCT imaging reveals anatomical defects associated with the XeroGel slow release vehicle that is confirmed by histology, and we note that the XeroGel vehicle is locally incompatible with histological analysis (Fig. 1F, F′). Histological analysis indicates that all regenerates (*n* = 5) displayed ventral tendon repair and the formation of attachment sites at the distal end of the regenerated bone (Fig. 1F′, arrowhead). BSA‐treated digits show truncation at the amputation plane, with no evidence of distal elongation (Fig. 1G, G′). Neither treatment group exhibited hair follicle regeneration (Fig. 1E′, F′). Statistical analysis confirms that BMP2‐treated digits achieve pre‐amputation bone length by 21 DPI (Fig. 1H, *P* ≤ 0.0001).

**Figure 2:**
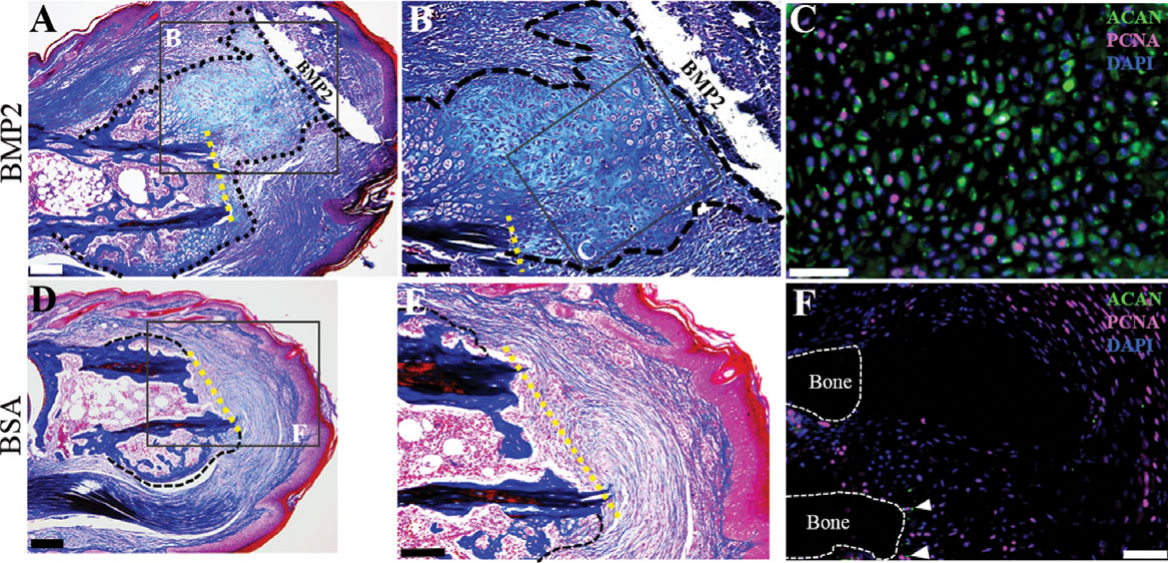
BMP2‐induced regeneration is mediated by a cartilaginous distal callus. (A)−(C) Serial sections of a representative BMP2‐treated sample at 8 DPI. (A) Mallory trichrome staining illustrating the cartilaginous distal callus in close association with the BMP2 XeroGel. The amputation plane is shown as the yellow dashed line. (B) Inset region of (A). Higher magnification view of the cartilaginous distal callus. (C) Inset region of (B). Double immunostaining for ACAN and PCNA show proliferating chondrocytes within the distal callus. Sample counterstained with DAPI. (D)−(F) Serial sections of a representative BSA‐treated sample at 8 DPI. (D) Mallory staining showing BSA control digits exhibit the characteristic peripheral callus but does not show chondrogenesis distal to the amputation plane, shown as the yellow dashed line. (E) Inset region of (D). Fibrotic tissue caps the distal end of the amputated P2 bone. (F) Double immunostaining for ACAN and PCNA shows few immunopositive chondrocytes (arrowheads), with proliferation localized to the fibrotic region. Sample counterstained with DAPI. (A)–(F) Distal is to the right, dorsal is to the top. Scale bars: (A), (D) 200 μm; (B), (E) 100 μm; (C), (F) 50 μm

To investigate the mechanism of BMP2‐induced regeneration, we carried out detailed histological and immunological analyses at 8 DPI. At 8 DPI, we observe a large mass of cells that formed a cartilaginous callus distal to the amputation plane associated with the BMP2 XeroGel vehicle (Fig. 2A, B). The cartilaginous distal callus is contiguous with, and appears to be an expansion of, the peripheral stump callus (Fig. 2A, B). Double immunostaining for the general cartilage marker Aggrecan (ACAN) and proliferating cell nuclear antigen (PCNA) shows proliferating chondrocytes localized throughout the distal callus (Fig. 2C). After BSA treatment, digits displayed no cartilaginous distal callus formation, instead exhibiting densely packed fibrous tissue capping the bone stump (Fig. 2D, E). Double immunostaining for ACAN and PCNA shows proliferating cells associated with the fibrotic response and few ACAN positive cells after BSA treatment (Fig. 2F, arrowheads). In summary, these data support the conclusion that BMP2 stimulates a regeneration response that is mediated by the formation of an intermediate cartilaginous callus that forms distal to the amputation plane and contiguous with the peripheral callus. Thus, it appears that BMP2 stimulation enhances the P2 amputation tissue repair response by directing callus formation distal to the bone stump.

### 2.2 Periosteum removal inhibits BMP2‐induced P2 regeneration

**Figure 3:**
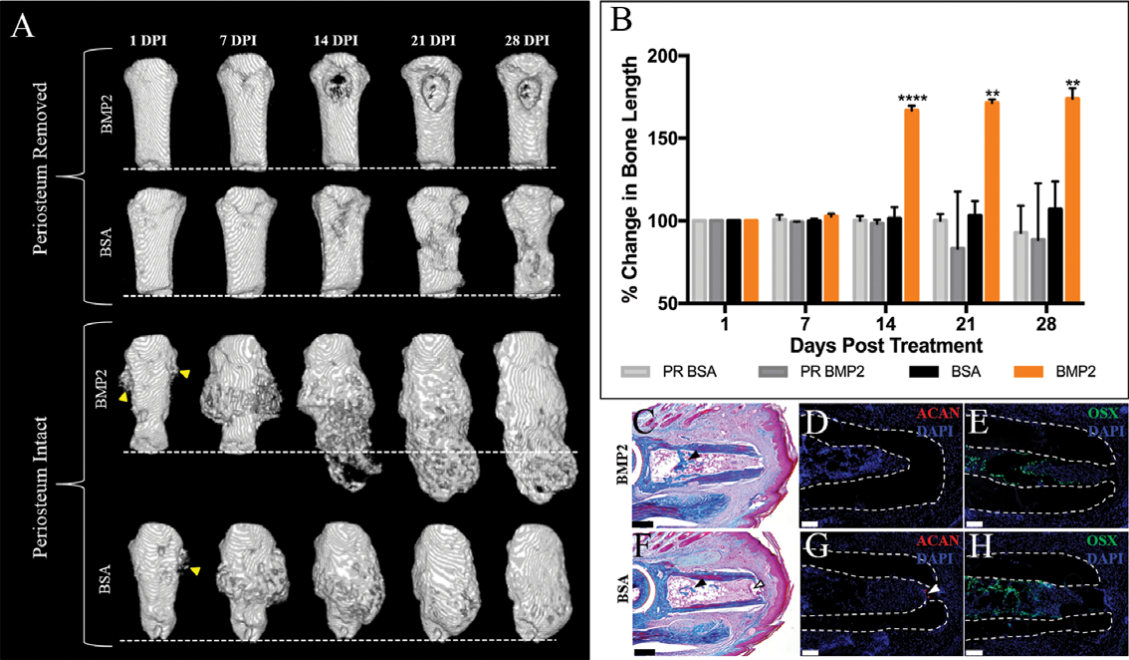
BMP2‐induced regeneration is inhibited in P2 digits lacking periosteum. (A) Representative μCT 3D renderings of periosteum removed or periosteum intact (sham) P2 digits treated with BMP2 or BSA XeroGel. Peripheral callus formation is not observed in BMP2‐ or BSA‐treated digits lacking the periosteum, and BMP2 treatment does not induce P2 regeneration in the absence of the periosteum. Peripheral callus formation is evident at 1 DPI (arrowheads) in BMP2‐ or BSA‐treated samples with intact periosteum, and distal callus formation is evident by 14 DPI in BMP2‐treated digits with intact periosteum. (B) Bone length changes over 4 weeks, normalized to 1 DPI, show no BMP2‐induced increase in bone length in digits lacking periosteum (ANOVA, ±SEM, **P ≤ 0.01, ****P ≤ 0.0001). (C)–(E) Serial sections of a representative BMP2‐treated digit lacking periosteum at 8 DPI. (C) Mallory trichrome staining showing no peripheral callus formation or distal callus formation. Bone formation is evident within the endosteal/marrow space (arrowhead). (D) ACAN immunostaining confirms no cartilage is present in digits lacking the periosteum. (E) OSX immunopositive cells are localized to the endosteal/marrow region. Samples counterstained with DAPI. (F)–(H) Serial sections of a representative BSA‐treated digit lacking periosteum at 8 DPI. (F) Mallory trichrome staining illustrating no peripheral callus formation or distal callus formation. Endosteal/marrow bone formation is evident (closed arrowhead), and residual scab is present within the marrow space of the distal bone stump (open arrowhead). (G) Immunostaining for ACAN shows no immunopositive cells distal to the amputation plane or associated with the periosteum. Arrowhead denotes background fluorescence associated with the residual scab. (H) OSX immunostaining is localized to the endosteal/marrow region. Samples counterstained with DAPI. (A) Distal is to the bottom. (C)–(H) Distal is to the right, dorsal is to the top. Scale bars: (C), (F) 200 μm; (D), (E), (G), (H) 50 μm

The periosteum is required for P2 peripheral callus formation (Dawson et al., 2016). To investigate the role of periosteal tissue in induced regeneration, BMP2 (*n* = 4) or BSA (*n* = 4) XeroGel was implanted into 9 DPA digit stumps in which the P2 periosteum was removed at the time of amputation. In parallel, sham operated digits with intact periosteum were treated with BMP2 (*n* = 3) or BSA (*n* = 4) XeroGel at 9 DPA. Changes in digit morphology and length were documented using μCT imaging for 4 weeks, with the representative sequential 3D renderings shown (Fig. 3A, B). Based on μCT imaging (Fig. 3A), P2 digits lacking the periosteum show no evidence of peripheral callus formation and no evidence of an induced BMP2 regeneration response. At all stages BMP2‐ and BSA‐treated digit amputations responded in a similar manner, thus indicating that the periosteum is required for BMP2‐induced regeneration. In the absence of the periosteum there is no callus formation and between 14 and 28 DPI there is evidence of enhanced bone degradation in both BMP2 and BSA treatments. On the other hand, sham operated digits display periosteal expansion similar to control digits that were simply amputated (compare Fig. 3A to Fig. 1C). At 14 DPI, distal elongation of the digit stump bone is evident in BMP2‐treated digits but not in BSA control digits (Fig. 3A, B, *P* ≤ 0.0001).

To assess for cartilaginous growth in response to BMP2 or BSA treatment in periosteum‐removed digits, we harvested treated digits at 8 DPI and performed detailed histological and immunological analysis. BMP2‐ or BSA‐treated digits show no cartilaginous or boney growth adjacent to the periosteum or distal to the amputation plane, yet exhibit boney growth within the endosteal/marrow space (Fig. 3C, F). Accordingly, immunostaining for ACAN revealed no positive chondrogenic signal in serial sections of BMP2‐ or BSA‐treated digits (Fig. 3D, G). Analogous to the histological findings, immunostaining using the osteoblast marker Osterix (OSX) showed immunopositive cells within the endosteal/marrow region of the BMP2‐ or BSA‐treated digits, yet we did not observe positive signal adjacent to the external bone surface or distal to the amputation plane (Fig. 3E, H). Our previous cell lineage studies showed that the endosteum/marrow space of P2 contributed osteoblasts in response to injury, congruent with our findings here (Dawson et al., 2016). Therefore, it appears that P2 endosteal/marrow derived osteoblasts are not the target of BMP2 treatment and furthermore cannot compensate for the periosteal tissue in induced regeneration. Taken together, these data provide evidence that the periosteal tissue is required for BMP2‐induced P2 regeneration.

### 2.3 Regeneration window

**Figure 4:**
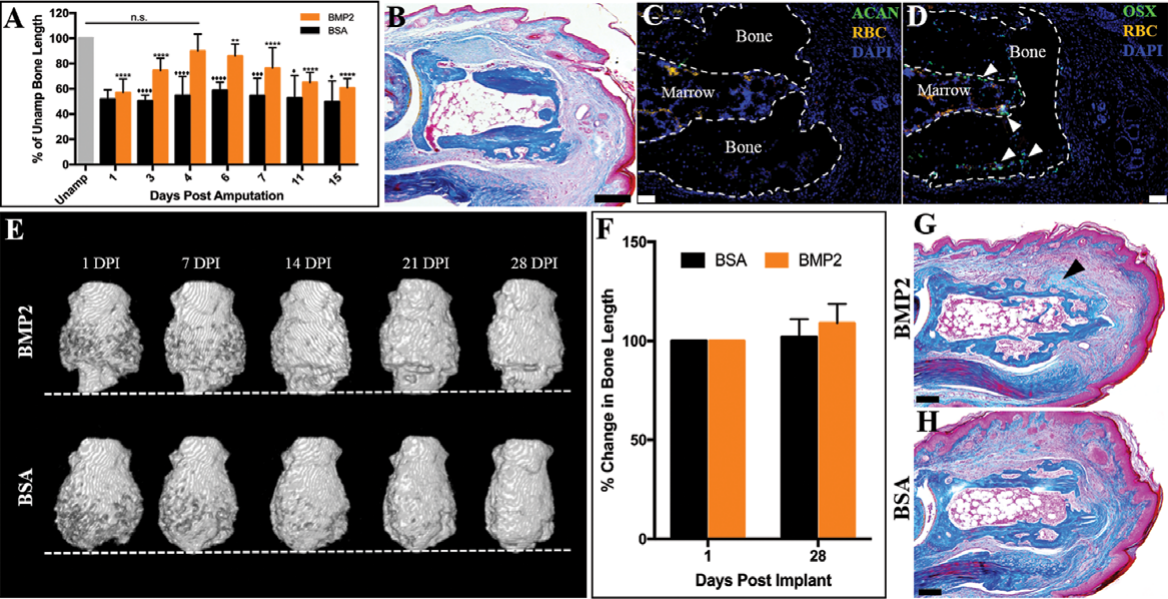
P2 wound maturation attenuates BMP2‐induced regeneration. (A) Neonate P2 skeletal length is restored after BMP2 treatment at 4 DPA, the approximate time of wound closure. BMP2 treatment prior to or after wound closure attenuates the regeneration response (ANOVA, ±SEM, *P or ♦*P* ≤ 0.05, **P ≤ 0.01, ***P or ♦♦♦*P* ≤ 0.001, ****P or ♦♦♦♦*P* ≤ 0.0001; asterisks denote significance in BMP2‐treated digits compared to BMP2 treatment at 4 DPA; diamonds denote significance between BMP2 and BSA treatment). (B)–(D) Serial sections of a representative 24 DPA P2 digit. (B) Mallory trichrome staining showing the cartilaginous peripheral callus has been completely remodeled to boney tissue. (C) Immunohistochemistry for ACAN shows no immunopositive cells localized to the peripheral callus at 24 DPA. (D) OSX immunostaining shows osteoblasts localized to the endosteal/marrow region and within the boney peripheral callus (arrowheads). Samples counterstained with DAPI. (E), (F) Representative μCT 3D renderings of amputated P2 digits treated with BMP2 at 24 DPA. The amputation plane is shown as the dashed line. Peripheral callus formation is observed by 24 DPA. BMP2 does not function to induce regeneration when implanted at 24 DPA, resulting in no enhancement of bone length from 1 to 28 DPI (*t* test, ±SEM, *P* > 0.05, no significance). (G) Mallory trichrome staining of a representative 24 DPA BMP2‐treated digit harvested at 8 DPI showing cartilage associated with the peripheral callus (arrowhead), not distal to the amputation plane. (H) Mallory trichrome staining of a representative 24 DPA BSA‐treated digit harvested at 8 DPI illustrating that the peripheral callus has been completely replaced with boney tissue and no evidence of distal elongation. RBC, red blood cells. (B)–(D), (G), (H) Distal is to the right, dorsal is to the top. (E) Distal is to the bottom. Scale bars: (B), (G), (H) 200 μm; (C), (D) 50 μm

BMP2‐induced regeneration of the neonate P2 digit is an established model of induced mammalian regeneration (Lee et al., 2013; Yu et al., 2012). Using this neonatal model, we explored whether the potential to respond to BMP2 changes as the amputation wound matures, thus inquiring if the efficacy of induced regeneration is modulated at different stages of wound healing. BMP2 treatment of the neonatal digit at 4 DPA, the approximate time of wound closure, restores the P2 skeletal length (Yu et al., 2012). BMP2‐induced regeneration of the neonate digit is attenuated with treatment prior to wound closure or treatment at later stages of wound healing (Fig. 4A, *n* ≥ 7). Treatment at 7 DPA results in approximately half of the skeletal restoration observed at 4 DPA (Fig. 4A, *P* ≤ 0.0001), while digits treated at 11 and 15 DPA show little enhancement of regeneration compared to BSA (Fig. 4A, *P* ≤ 0.05). These data suggest that the amputation wound is dynamic and undergoes changes in regenerative potential associated with wound maturation, and thus identifies a “regeneration window” with respect to BMP2 responsiveness during the wound healing process.

To investigate the regeneration window in the adult digit amputation model, digits were amputated at the P2 level and allowed to heal for 24 days, over 2 weeks post wound closure. By 24 DPA, the P2 cartilaginous peripheral callus had been completely replaced with woven bone (Fig. 4B). Immunostaining for ACAN confirmed the absence of chondrocytes at 24 DPA (Fig. 4C), and immunostaining for OSX revealed positive signal within the central marrow region and in the woven bone of the peripheral callus (Fig. 4D, arrowheads). At 24 DPA, BMP2 or BSA XeroGel (*n* = 4) was implanted into the distal digit tip between the stump bone and the apical epidermis. BMP2‐induced regeneration was quantified using μCT scans over the next 4 weeks. μCT renderings of 24 DPA BMP2‐ or BSA‐treated digits show that, by 1 DPI, both treatment groups had previously undergone peripheral callus formation and ossification (Fig. 4E). At 14, 21, and 28 DPI, time points typically associated with a robust BMP2‐induced regeneration response, no distal elongation of bone in response to BMP2 treatment was observed. Rather, continued remodeling of the peripheral callus in BMP2‐ and BSA‐treated digits was apparent (Fig. 4E). Statistical analysis confirms no significant change in bone length from 1 DPI to 28 DPI and no significant differences between the two treatment groups (Fig. 4F, *P* > 0.05). 24 DPA BMP2‐treated and BSA control digits were analyzed at 8 DPI to assess for cartilaginous distal callus formation (Fig. 4G, H). 8 DPI 24 DPA BMP2‐treated digits displayed some small clusters of peripheral chondrocytes (arrowhead); however, we did not observe the cartilaginous distal callus that typifies induced regeneration (Fig. 4G). There was no indication of cartilaginous growth in 8 DPI 24 DPA BSA control digits (Fig. 4H). These findings point to a regeneration window of BMP2 responsiveness during amputation healing of the adult digit and, coupled with the periosteal requirement for induced regeneration, suggest that this window may be linked to dynamic changes associated with the chondrogenic response of the injured periosteal tissue.

**Figure 5:**
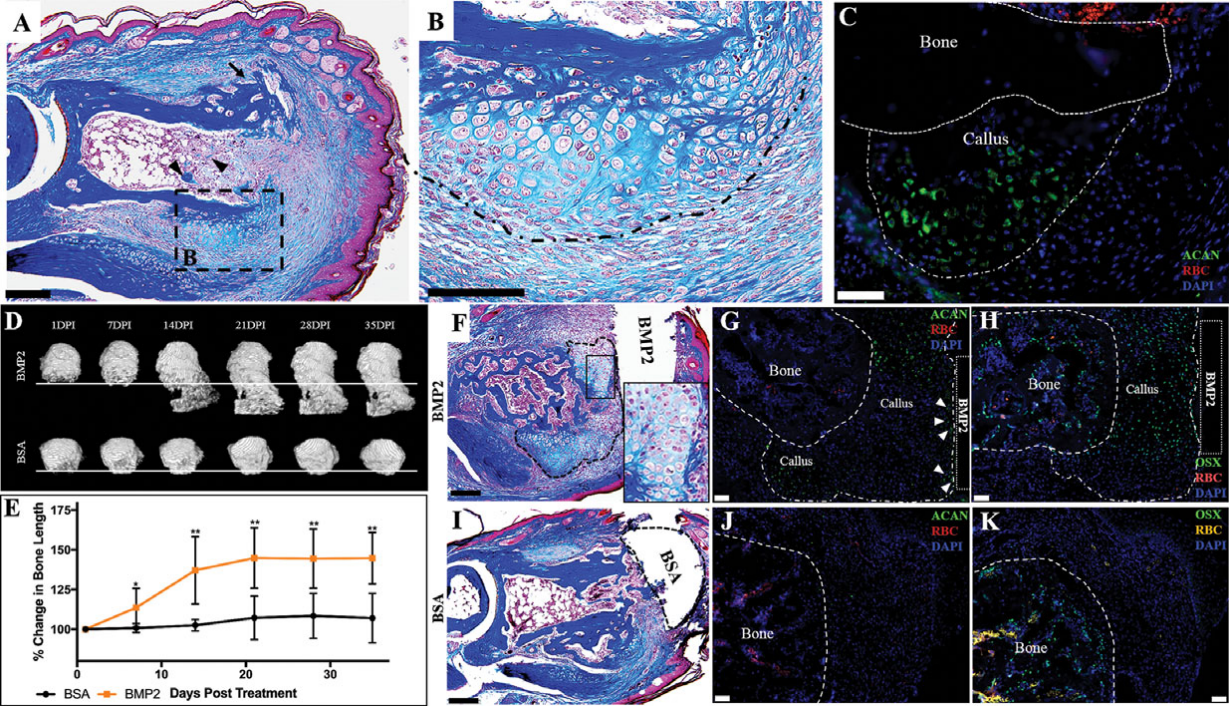
BMP2 induces regeneration of the re‐injured mature P2 bone stump. (A)–(C) Serial sections of a representative 24 DPA reamputated P2 digit at 9 DPA. (A) Mallory trichrome staining showing peripheral callus formation is reinitiated after stump re‐injury. (B) Inset illustrating that peripheral callus chondrogenesis develops external to the pre‐existing peripheral callus. (C) The peripheral callus associated with P2 re‐injury tests immunopositive for ACAN. Sample counterstained with DAPI. (D) Representative μCT renderings illustrate that BMP2 induces regeneration of mature P2 wounds after re‐injury. The regeneration response, evident by 14 DPI, is mediated by the formation of a distal callus, whereas BSA‐treated digits show no distal callus formation or associated bone elongation. The line indicates the amputation plane. (E) Statistical analysis of bone length measurements over 35 days, normalized to 1 DPI, show that BMP2 induces a significant increase in bone length compared to re‐injured digits treated with BSA (*t* test, ±SEM, *P ≤ 0.05, **P ≤ 0.01). (F)–(H) Serial sections of a representative BMP2‐treated digit at 10 DPI. (F) Mallory trichrome staining shows chondrogenesis associated with the BMP2 XeroGel. Inset displaying high magnification of chondrocytes. Distal callus is outlined. (G) ACAN immunostaining is localized to the boundaries of the distal callus, while (H) OSX immunostaining is localized to the central region of the distal callus, in areas not immunopositive for ACAN staining. Samples counterstained with DAPI. (I)–(K) Serial sections of a representative BSA‐treated digit at 10 DPI. (I) Mallory trichrome staining of BSA‐treated digits show peripheral callus with no evidence of distal callus formation. (J) BSA‐treated digits test negative for ACAN immunostaining. (K) OSX immunostaining is localized to the bone stump (outlined). Samples counterstained with DAPI. (A)–(C), (F)–(K) Distal is to the right, dorsal is to the top. (D) Distal is to the bottom. Scale bars: (A), (F), (I) 200 μm; (B) 100 μm; (C), (G), (H), (J), (K) 50 μm

Working under the general hypothesis that active chondrogenesis is a target for exogenous BMP2 we carried out re‐injury studies to determine if a healed amputation wound (BMP2 unresponsive) could be stimulated to become BMP2 responsive. In other words, can the regeneration window be reopened? P2 digits were amputated and allowed to heal for 24 days. At 24 DPA, the apical wound epidermis was surgically removed and the stump bone was re‐injured by removing the apical bone to expose the bone marrow. The re‐injury was allowed to heal conservatively and at 9 days post re‐injury the digits were harvested and assayed for periosteal chondrogenesis using histology and immunohistochemistry (Fig. 5A−C). There is histological evidence that the 24 DPA re‐injured digit displayed characteristics of an initial healing response (i.e. the presence of woven bone in the peripheral callus [arrow] and bone nodules in the bone marrow [arrowheads]) (Fig. 5A), as well as new cartilaginous growth associated with the peripheral callus that formed external to the initial periosteal response (Fig. 5B, callus outlined). ACAN immunostaining is localized to the margin of the new peripheral callus, but not distal to the amputation plane; thus the re‐injury healing response recapitulates the cartilaginous peripheral response of the original amputation injury (Fig. 5C).

To test if healed and re‐injured amputations can be induced to regenerate, the 24 DPA re‐injured P2 bone stump was treated with BMP2 (*n* = 8) or BSA (*n* = 5) XeroGel 9 days after re‐injury. Implants were placed between the newly formed wound epidermis and the re‐injured bone stump. Digit regeneration was quantified using μCT for 5 weeks. μCT 3D renderings at 1 DPI indicate that the BMP2‐treated and BSA control digits have undergone prior periosteal bone remodeling (Fig. 5D). Statistical analysis of bone length changes indicates a small but significant average increase in digit length in BMP2‐treated samples at 7 DPI (Fig. 5E, *P* ≤ 0.05). By 15 DPI, 3D renderings show extensive distal boney callus formation and associated stump elongation in response to BMP2 treatment (Fig. 5D, E, *P* ≤ 0.01). At 21 DPI, the regenerated bone shows evidence of continued distal bone growth and remodeling, while BSA‐treated digits show little change (Fig. 5D, E, *P* ≤ 0.01). At 28 and 35 DPI, BMP2‐treated digits show signs of bone remodeling and have essentially terminated distal growth, yet maintained the length of the regenerated bone (Fig. 5D, E, *P* ≤ 0.01).

To determine if the re‐injury regeneration response was mediated by a distal cartilaginous callus, we harvested samples at 10 days post BMP2 or BSA treatment for histological and immunological analysis. At 10 DPI, a distal callus (outlined) has formed associated with the BMP2 source and shows evidence of chondrogenesis (Fig. 5F, inset). Immunostaining for ACAN shows chondrocytes in the distal callus but largely associated with the boundaries of the callus and not present in the central region of the distal callus (Fig. 5G). ACAN positive cells are also observed directly associated with the BMP2 XeroGel implant (Fig. 5G, arrowheads). In control BSA‐XeroGel‐treated digits ACAN immunopositive chondrocytes were not observed distal to the amputation plane (Fig. 5J). While chondrocytes were not observed in the central region of the distal callus, this region stained positive for the osteoblast marker OSX (Fig. 5H) indicating that the central region of the distal callus was ossifying. OSX expression was also observed in the peripheral callus and bone marrow proximal to the amputation plane in both BMP2‐ and BSA‐treated digits (Fig. 5H, K). The spatial organization of chondrocytes and osteoblasts within the distal callus was distinct from BMP2‐induced regenerates from an initial amputation which raises the possibility that the response to re‐injury may not be identical. Nevertheless, these studies support the conclusion that the regeneration window of previously healed amputation injuries has the potential to be “reopened” by re‐injury, thus reinitiating the repair response, and the cells of the wound are able to mount a regenerative response to BMP2 treatment.

## 3 DISCUSSION

The mouse digit is a unique model system to investigate regeneration; not only is it proof of concept that mammals can indeed regenerate certain structures, such as after distal P3 amputation, it is also a system in which to assess regenerative failure and induced regeneration of P2. By focusing on the healing response following P2 level amputations, a dynamic bone and soft tissue repair response was characterized (Dawson et al., 2016). The periosteum of the amputated bone reacts in a manner similar to fracture healing by forming a transient proliferative chondrogenic callus that builds new bone peripherally only to have the new bone remodeled back to the original stump morphology. In the current study we have found that targeted treatment with BMP2 can modify this periosteal response to create a chondrogenic distal callus that facilitates the regeneration of new bone to restore the amputated P2 bone. While imperfect, the regenerated new bone is anatomically integrated with the stump bone, possesses a bone marrow that is contiguous with the stump marrow and, after remodeling, displays anatomical features of the amputated P2 bone. These structural similarities are suggestive that the BMP2‐induced regenerated bone is appropriately patterned, which is consistent with previous studies on BMP2‐induced regeneration in neonates (Yu et al., 2012). Further, we find evidence that some of the surrounding soft tissue, i.e. tendon fibers, undergo repair in conjunction with the newly forming bone. These findings support the general conclusion that targeted growth factor treatment to modify/extend intrinsic repair mechanisms can be effective for enhancing regeneration following amputation. Stimulating skeletal regeneration by distraction osteogenesis also involves enhancing intrinsic repair mechanisms (Ilizarov, 1989); however, distraction osteogenesis induces the regeneration of new bone by direct ossification (Dhaliwal, Kunchur, & Farhadieh, 2016; Hvid, Horn, Huhnstock, & Steen, 2016) and so it is distinct from BMP2‐induced regeneration which stimulates formation of new bone de novo by endochondral ossification.

BMP2 stimulates segment‐specific P2 regeneration by inducing the formation of a distal chondrogenic callus that is contiguous with the peripheral callus that forms in response to simple amputation. Since BMP2‐induced regeneration is inhibited when the periosteum is surgically removed, the evidence suggests that periosteal cells of the stump play a role in the induced regeneration response. The periosteum is critical for callus formation in fracture repair and amputation healing (Colnot, 2009; Dawson et al., 2016); thus the formation of the peripheral chondrogenic callus is a prerequisite for the induced regeneration response. Periosteal chondrogenesis has been linked to the upregulation of BMP2 at the fracture site (Tsuji et al., 2006), periosteal‐derived BMP2 is required for chondrogenic induction after bone injury (Wang et al., 2011), and periosteal cells form cartilage in response to BMP2, while BMP2 functions to induce osteogenesis (Minear et al., 2010) or to have no effect on endosteal cells (Yu, Lieu et al., 2010). These findings support the conclusion that BMP2 is a key endogenous factor controlling the periosteal response to injury and that treatment with BMP2 enhances an endogenous response by extending the period of BMP2 signaling.

In neonatal digits, BMP2‐induced P2 regeneration does not involve callus formation, but induces the formation of a distal endochondral ossification center (Yu et al., 2012) that is similar to the distal callus observed in the current study in that they both mediate endochondral ossification. In addition to induced proliferation, BMP2 enhances cell recruitment to the wound environment in the neonate digit model by inducing SDF‐1α production which recruits CXCR4 positive cells to the amputation wound (Lee et al., 2013). SDF‐1α/CXCR4 signaling has been implicated in cell recruitment in the regenerating zebrafish fin (Dufourcq & Vriz, 2006), mouse digit tip regeneration (Lee et al., 2013), fracture repair (Kitaori et al., 2009), and BMP2‐induced ectopic bone formation (Otsuru, Tamai, Yamazaki, Yoshikawa, & Kaneda, 2008). In BMP2‐treated adult P2 amputations, the expression of CXCR4 by cells of the induced distal callus is suggestive that a similar mechanism acts to recruit cells to the distal digit stump (Fig. S1). These findings support a model in which BMP2 also functions indirectly to recruit cells that form the distal callus and, by doing so, provides a distal signal that organizes the induced regeneration response.

Successful regeneration involves a series of stepwise interactions involving regeneration‐competent cells that culminate in the functional replacement of amputated structures (Endo, Bryant, & Gardiner, 2004; Simkin, Sammarco, Dawson, Tucker et al., 2015; Tanaka, 2016). With this in mind, regenerative failure can result from a defect in the absence of regeneration‐competent cells and/or one or all of the stepwise interactions. In any regeneration‐incompetent injury model, induced regeneration indicates that responding cells have the potential to mount a regeneration response, yet fail to do so under normal conditions. Thus, the success of P2 regeneration indicates that regenerative failure stems from a restriction in BMP2 signaling and is not linked to an inherent inability of cells to respond to a regeneration‐permissive wound environment. The results from a growing number of studies including matrix implantation (Agrawal, Kelly et al., 2011), histolytic degradation (Agrawal et al., 2012; Sammarco et al., 2015) and cell transplantation between regeneration‐incompetent and regeneration‐competent tissues (Wu et al., 2013) all support the general conclusion that P2 regenerative failure is causally linked to defects in the wound environment and is not limited by the availability of responsive cells. We also provide evidence that the wound environment is dynamic with respect to BMP2 responsiveness, transitioning from a responsive phase to a non‐responsive phase that reflects a regeneration window during the healing response. A similar transitioning of the wound environment has been observed associated with BMP2 treatment in segmental bone defects (Hussein et al., 2012). The dynamics of a regeneration window are expected to be specific for the inducing agent and probably modify the responsiveness of wound cells to other regeneration‐inducing treatments. The important conclusion is that the wound site is dynamically changing and therefore predicted to be differentially responsive to regeneration‐inducing agents. While this conclusion seems daunting with respect to understanding regenerative competency in mammals, the demonstration that a regeneration‐permissive wound environment can be restored by re‐injury provides encouragement that cells can regain regenerative capabilities long after an unsuccessful repair response. This indicates that a failed regenerative response does not modify the regenerative potential of cells at the wound site; they are able to initiate a healing response that can transition into a period of responsiveness to a regeneration‐inducing agent such as BMP2. This conclusion has important clinical implications for potentially reactivating regenerative potential in amputated limbs after stump healing is completed.

Broadly speaking, regeneration is divided into categories designed to organize and contrast the various regenerative and reparative responses that occur in nature. Epimorphic regeneration, the designation within which amphibian limb and mammalian distal digit tip regeneration belong, is mediated by a proliferative blastema, a transient undifferentiated structure that mediates the restoration of the amputated structure (Carlson, 2007). The digit blastema forms by recruitment of cells from multiple tissue types to create a proliferative heterogeneous population that redifferentiates in a lineage‐specific manner (Fernando et al., 2011; Lee et al., 2013; Lehoczky et al., 2011; Rinkevich et al., 2011; Simkin, Sammarco, Dawson, Schanes et al., 2015; Wu et al., 2013). Conversely, skeletal regeneration in response to fracture is defined as a tissue repair response not mediated by blastema formation (Carlson, 2007). Yet, like the blastema, the chondrogenic callus that forms in fracture healing and digit amputation is proliferative, displays similar recruitment characteristics, and is macrophage dependent (Miura et al., 2015, Simkin et al., 2017). What distinguishes the callus from a blastema is the differentiative state and relative homogeneity of composite cells (chondrogenic), and the fact that fracture healing is a nerve‐independent process (Miura et al., 2015) which may be linked to the lack of cellular heterogeneity. The homogeneity of cells that make up the callus is strikingly similar to the cellular composition of the deer antler blastema that mediates their annual regeneration (Kierdorf, Kierdorf, & Szuwart, 2007). Based on the results presented here, the chondrogenic callus displays blastema characteristics and possesses considerable regenerative potential when properly stimulated. The distinction between a blastema and a callus is perhaps best considered in light of the idea that the blastema mediates intersegmental regeneration (i.e. the formation of segments) and the callus mediates intrasegmental regeneration (i.e. the formation of structures within a segment) (Satoh, Cummings, Bryant, & Gardiner, 2010). This delineation helps us to understand the relationship between epimorphic and tissue‐specific regenerative responses.

## 4 MATERIALS AND METHODS

### 4.1 Animals and surgery

Adult 8‐week‐old female C57Bl/6 mice were purchased from Charles River (Wilmington, MA) or bred in house at the Texas Institute for Genomic Medicine. Mice were anesthetized with isoflurane for all surgical procedures. Digits were amputated at the level of the second phalangeal element (P2; Fig. 1A, B) as described (Dawson et al., 2016). Digits used in the P2 re‐injury study were initially amputated at the P2 level and were allowed to heal for 24 days. At 24 DPA the apex of the healed digit stump skin was removed and the stump bone was re‐injured by removing the distal cap of healed bone that encloses the bone marrow (Dawson et al., 2016). After re‐injury, the stump wound was allowed to heal conservatively. For periosteum removal studies, the digits were amputated at P2 and the periosteum was mechanically removed as previously described (Dawson et al., 2016). The distal amputation wound was closed with Dermabond (Ethicon, Somerville, NJ). Sham control digits were treated identically, but the periosteum was not removed. All animal use and techniques were compliant with the standard operating procedures of the Institutional Animal Care and Use Committees at Tulane University and the College of Veterinary Medicine and Biomedical Sciences at Texas A&M University.

### 4.2 BMP2 and BSA treatment groups

Various combinations of P2 amputation(s) and BMP2 (R&D Systems Inc., Minneapolis, MN) or BSA (Sigma‐Aldrich Co., St Louis, MO) treatment were performed in this study: (**1**) BMP2 treatment after wound closure (9 DPA), (**2**) BMP2 treatment after stump healing (24 DPA), and (**3**) BMP2 treatment after stump healing, re‐injury of the P2 stump and conservative healing (33 DPA). Hindlimb digits 2 and 4 were amputated at the P2 level and allowed to heal conservatively. At the time of treatment, a slow release implant containing either BMP2 (0.5 μg/μL) or BSA (0.1%) was inserted into a surgically created pocket between the wound epidermis and the P2 bone stump, and the wound was closed using Dermabond. The slow release vehicle used was a sol−gel silica‐based porous glass (XeroGel, Entellus Medical, Plymouth, MN). XeroGel is a hemostatic packing that uses a unique blend of polyethylene glycol and chitosan to separate tissues and prevent adhesions between mucosal surfaces, and functions as a slow release vehicle for embedded growth factors for up to 63 days (Agrawal & Sinha, 2016; Nicoll, Radin, Santos, Tuan, & Ducheyne, 1997; Santos, Radin, & Ducheyne, 1999). BMP2 or BSA containing XeroGel was prepared as described by Santos et al. (1999). Protein containing XeroGel solution was aliquoted into 1.0 μL drops, air‐dried at room temperature, and stored at −20°C until use.

### 4.3 Histology and immunohistochemistry

Tissue processing has been previously described (Dawson et al., 2016). Briefly, digits were fixed in buffered zinc formalin (Z‐Fix, Anatech Ltd, Battle Creek, MI; 24−96 h), decalcified in Decalcifier I (Surgipath, Leica Biosystems, Richmond, IL; 24 h), paraffin embedded, and serially sectioned at 4.5 μm. Digits were stained with Mallory trichrome to illustrate general histology. Samples were imaged using an Olympus BX60 microscope and Olympus DP72 camera, with image processing performed using the DP2‐BSW software (Olympus America Inc., Center Valley, PA). For immunostaining, paraffin sections were deparaffinized and washed in Tris buffered saline with Tween® 20 (Sigma‐Aldrich Co.). Antigen retrieval was carried out using either proteinase K (Dako, Carpinteria, CA; 10 mg/ml, 37°C, 12 min), or heat retrieval (Dako; pH 6 citrate buffer, 90°C, 20 min). Slides were treated in Protein Block Solution (Dako; 1 h, room temperature). Immunostaining for PCNA was performed using heat retrieval, monoclonal anti‐mouse antibody (Abcam, Cambridge, UK; ab29**,** 1:2000 dilution), and the Alexa Fluor 647 goat anti‐mouse IgG secondary antibody (Invitrogen, Carlsbad, CA; A21235**,** 1:500 dilution). Cartilage immunostaining was performed using heat retrieval, rabbit anti‐mouse Aggrecan polyclonal antibody (EMD Millipore, Billerica, MA; AB1031, 1:300 dilution) and either the Alexa Fluor 568 goat anti‐rabbit IgG or the Alexa Fluor goat anti‐rabbit 488 IgG secondary antibody (Invitrogen; A11011, A11008, 1:500 dilution). Immunostaining for CXCR4 was performed using proteinase K, rat anti‐CXCR4 antibody (R&D Systems; MAB21651, 1:500 dilution) and the Alexa Fluor goat anti‐rat 568 IgG secondary antibody (Invitrogen; A11077, 1:500 dilution). Immunostaining for osteoblasts was performed using heat retrieval, rabbit anti‐Osterix, SP7 polyclonal antibody (Abcam; ab22552, 1:400 dilution) and the Alexa Fluor goat anti‐rabbit 488 IgG secondary antibody (Invitrogen; A11008, 1:500 dilution). Slides were imaged using the Olympus BX61 fluorescence deconvolution microscope utilizing Slidebook software (Intelligent Imaging Innovations Inc., Denver, CO).

### 4.4 Micro‐computed tomography (μCT) scans, length, and image processing

P2 digits were scanned at weekly intervals beginning at 1 day post BMP2 or BSA implant (DPI) for 5 total weeks, with some treatment groups receiving an end‐point scan at 160 DPI. Scans were performed using the vivaCT 40 (SCANCO Medical, Wayne, PA) as previously described (Dawson et al., 2016; Fernando et al., 2011). P2 digits were scanned at a voxel size of 10.5 μm and energy of 45 kVp; 1000 projections per 180^o^ were captured at 380 msec using continuous rotation. μCT images were saved as a series of dicom files, and the dicom sequences were uploaded to ImageJ. Using the BoneJ (Doube et al., 2010) (Version 1.2.1) Optimized Threshold Plugin for ImageJ, images were segmented and converted to 3D renderings, and length measurements were quantified as previously described (Dawson et al., 2016).

## Supporting information


**Figure S1**. Tissue section of the BMP2‐induced distal chondrogenic callus at 8 DPI, showing CXCR4 immunopositive cells localized throughout the callus and adjacent to the BMP2 XeroGel (outlined). Sample counterstained with DAPI. Scale bar 50 μm.
