# Supplementary figures and images for: The periosteal requirement and temporal dynamics of BMP2‐induced middle phalanx regeneration in the adult mouse
Source: Regeneration (Oxf). 2017 Aug 20;4(3):140–50. doi: 10.1002/reg2.81 (PMC5617898; doi:10.1002/reg2.81)

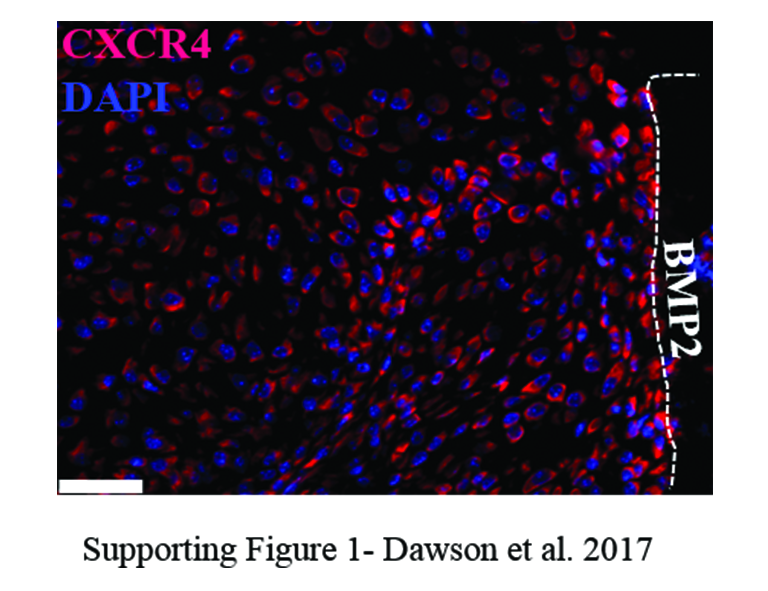

Supplement: Supplementary file 1 — Figure S1. Tissue section of the BMP2‐induced distal chondrogenic callus at 8 DPI, showing CXCR4 immunopositive cells localized throughout the callus and adjacent to the BMP2 XeroGel (outlined). Sample counterstained with DAPI. Scale bar 50 μm. [file REG2-4-140-s001.tif]
